# A feasibility study of goal-directed network-based real-time fMRI neurofeedback for anhedonic depression

**DOI:** 10.3389/fpsyt.2023.1253727

**Published:** 2023-12-05

**Authors:** Xiaoxia Wang, Xiaoyan Zhou, Jing Li, Yushun Gong, Zhengzhi Feng

**Affiliations:** ^1^Department of Basic Psychology, School of Psychology, Army Medical University, Chongqing, China; ^2^Chongqing City Mental Health Center, Southwest University, Chongqing, China; ^3^Department of Radiology, Southwest Hospital, Army Medical University, Chongqing, China; ^4^Department of Medical Equipment and Metrology, College of Biomedical Engineering, Army Medical University, Chongqing, China; ^5^School of Psychology, Army Medical University, Chongqing, China

**Keywords:** rt-fMRI neurofeedback, CEN/SN, prediction error, anhedonia, goal-directed system

## Abstract

Anhedonia is a hallmark symptom of depression that often lacks adequate interventions. The translational gap remains in clinical treatments based on neural substrates of anhedonia. Our pilot study found that depressed individuals depended less on goal-directed (GD) reward learning (RL), with reduced reward prediction error (RPE) BOLD signal. Previous studies have found that anhedonia is related to abnormal activities and/or functional connectivities of the central executive network (CEN) and salience network (SN), both of which belong to the goal-directed system. In addition, it was found that real-time functional magnetic resonance imaging (rt-fMRI) neurofeedback (NF) could improve the balance between CEN and SN in healthy individuals. Therefore, we speculate that rt-fMRI NF of the CEN and SN associated with the GD system may improve depressive and/or anhedonic symptoms. Therefore, this study (1) will examine individuals with anhedonic depression using GD-RL behavioral task, combined with functional magnetic resonance imaging and computational modeling to explore the role of CEN/SN deficits in anhedonic depression; and (2) will utilize network-based rt-fMRI NF to investigate whether it is feasible to regulate the differential signals of brain CEN/SN of GD system through rt-fMRI NF to alleviate depressive and/or anhedonic symptoms. This study highlights the need to elucidate the intervention effects of rt-fMRI NF and the underlying computational network neural mechanisms.

## Introduction

Anhedonia is a transdiagnostic psychiatric symptom typical of psychiatric disorders, such as depression, posttraumatic stress disorder (PTSD), and schizophrenia according to the Diagnostic and Statistical Manual of Mental Disorders, Fifth Edition (DSM-5), and is associated with an elevated risk of suicide and chronicity of the disease (1). The biological endophenotypes of anhedonia include reduced anticipation of upcoming rewards, reduced experienced pleasure during the reward presentation, and impaired reward learning (2). Specifically, reward learning (RL) is the ability to adapt to the contingencies between action and the reward feedback, with two systems working together: The habitual system supports routine responses to deal with regular situations, while the goal-directed (GD) system is sensitive to environmental change to flexibly adapt to unfamiliar situations. The deficits in goal-directed systems play an important role across the lifespan and in various mental disorders, such as subclinical depression, trauma-related disorders, addiction, and obsessive-compulsive disorder (OCD) (3–6). Specifically, goal-directed reward learning (GD-RL) could discriminate between depressed patients and healthy controls using a machine learning algorithm (Naive Bayes) and could predict the concurrent depressive symptoms and depression traits 1 year later (7). To sum up, deficits in the goal-directed system may contribute to depression during both the symptomatic and clinical phases. However, a translational gap still remains in the clinical imaging study of the goal-directed system (8).

Neurally, the goal-directed system may involve functional interactions between the central execution network (CEN) and the salience network (SN). The CEN, including the medial frontal gyrus (MFG), superior frontal gyrus (SFG), and the anterior cingulate cortex (ACC), especially the perigenual ACC (pgACC), is essential for external, goal-directed cognitive processes (9, 10). The SN, including the anterior insula (aINS), the dorsal anterior cingulate cortex (dACC), the amygdala (AMY), the substantia nigra/ventral tegmental area (SN/VTA), ventral striatum (VS), and thalamus, is responsible for detecting salient stimuli in the environment and allocating cognitive resources to facilitate goal-directed behavior (10, 11). SN may function as a gate to detect the salience of stimuli and co-activate task-relevant networks such as CEN to facilitate goal-directed behavior (12), through the shift between default mode network (DMN) and CEN (13). Previous studies showed global circuit–phenotype associations across clinical anxiety and depression, with distinct CEN (e.g., ACC and dlPFC) and SN (e.g., aINS and AMY) circuits (14). Therefore, abnormalities in the goal-directed system, and in particular the CEN/SN decoupling, may represent distinctive neural phenotypes of depression.

**Figure 1:**
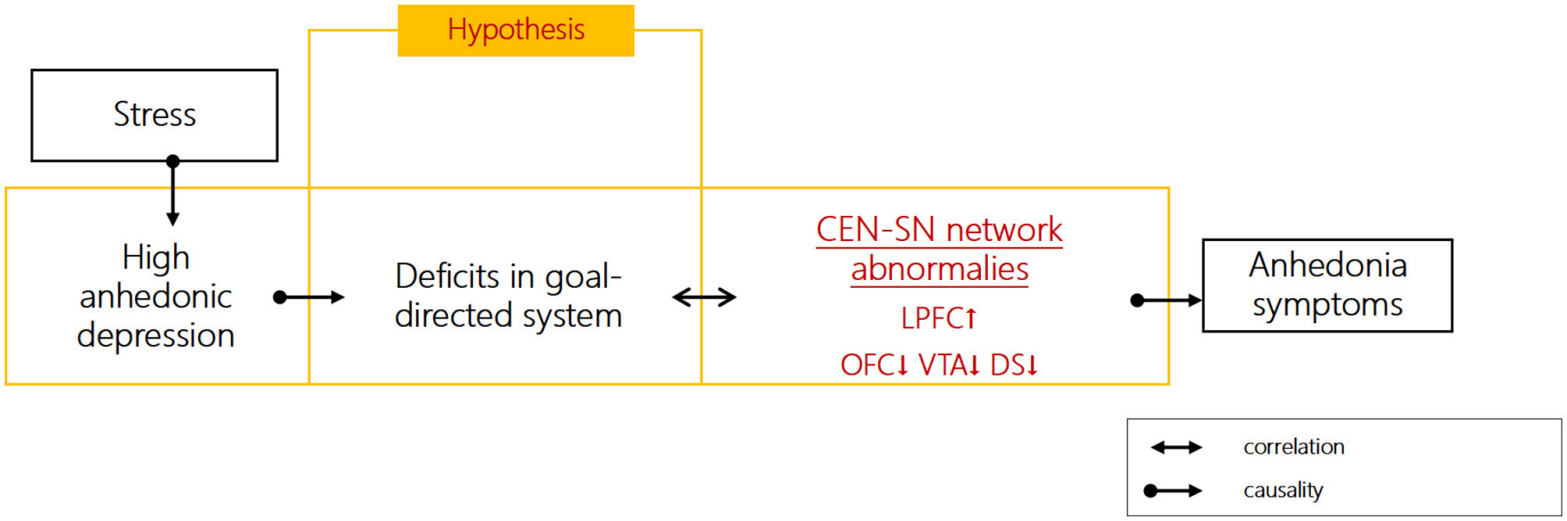
Hypothesized role of goal-directed function impairments in anhedonia symptoms of depression.

On the one hand, the dysfunctional networks of CEN and SN persist during depressive episodes. We previously found that depressed patients showed impaired goal-directed behavior, accompanied impairments in CEN, such as over-activation in the left lateral PFC (LPFC), as well as SN, such as under-activation in VTA, dorsal striatum (DS), and orbital frontal cortex (OFC) (7) (Figure 1), the last of which plays a critical role in controlling the transition between goal-directed and habitual behavior (15). The neural abnormalities in VTA during habitual learning do not normalize during remission (16). Further evidence suggested that enhanced resting-state functional connectivity (RSFC) between subregions of ACC (including pgACC and sgACC) and caudate could predict future anhedonia symptoms in adolescent depression (17, 18). Specifically, neural activation to uncertain rewards in ACC during reward learning tasks could predict anhedonia symptoms 1 year later when baseline anhedonia is controlled for (19). Collectively, these studies suggest that neural impairments in the CEN and SN within the goal-directed system are not only present during the acute phase of depression but also predict the course of the disease.

On the other hand, the stress-resilience model of depression provides a framework for understanding distinct subnetworks (CEN and SN) underlying anhedonic symptoms of depression. Emerging findings from functional abnormalities of higher functional connectivity between CEN (dlPFC and pgACC) and SN (striatum and VTA) during reward-related tasks were shown in depressed patients, as well as hypo-activation in CEN (pgACC and mOFC) and SN (VS/DS) (20). Anhedonia was associated with SN overactivity in the resting state, lack of SN activation in response to positive stimuli in task-related states, and SN overactivity in response to negative stimuli (21). Stress mediates the relationship between goal-directed reward learning and anhedonia (7). Stress may contribute to impaired goal-directed processes by enhancing the connectivities between CEN and SN circuits (e.g., dlPFC and amygdala/VS) (22). The resilience to stress involves the interactions among heightened CEN (PFC) and lessened SN (mesostriatal reward circuits) neural activities (23). Taken together, goal-directed behavior depends on the adaptive redistribution of neural resources between the CEN (↓) and SN (↓), and stress may lead to an imbalance between the CEN (↓) and SN (↓), as well as aberrant connectivity during reward processing, which may contribute to anhedonia symptoms.

Neurofeedback based on real-time functional magnetic resonance imaging (rt-fMRI) uses measured changes in brain activation to help participants regulate activity in selected regions or networks. During rt-fMRI neurofeedback, participants are provided with visually presented brain network activation information in real time (24). The rt-fMRI neurofeedback targeting the brain areas within SN involved in emotion processing (e.g., amygdala, insula, and striatum) decreased the anhedonic or depressive scores (25, 26). However, the effects of rt-fMRI neurofeedback (NF) based on CEN-SN on anhedonia to the best of our knowledge have not been investigated previously. Network-based NF can be used to recalibrate the balance between brain networks (27). It has been recommended that researchers use NF methods that consider neural response across regions (i.e., functional connectivity) (28). Recent studies have found that participants can flexibly regulate the balance between the CEN and SN through rt-fMRI neurofeedback which could promote resilience to stress (29). Therefore, the use of rt-fMRI neurofeedback to improve goal-directed reward learning is a potential intervention that involves the interaction between CEN and SN. Additionally, reward learning studies have typically adopted computational modeling during behavioral tasks to estimate expected value and reward prediction errors (RPEs) (20). Therefore, the current study was designed to adopt reward learning tasks (30) and computational modeling in anhedonic individuals to examine goal-directed brain network dynamics. Network-based rt-fMRI neurofeedback will be utilized to improve the balance of CEN/SN within the goal-directed brain network (Figure 1). The within-group changes of depressive symptoms after rt-fMRI NF training of goal-directed brain network will be examined to assess the effects of rt-fMRI NF intervention to improve depressive and/or anhedonic symptoms for depressed patients. The goal-directed RL, as well as CEN/SN changes (CEN-SN functional connectivity) at 3- and 6-month follow-ups, will also be examined to evaluate the long-term rt-fMRI neurofeedback training effects. This study may provide evidence to elucidate the effect of rt-fMRI NF based on the goal-directed system in alleviating depressive/anhedonic symptoms. The rt-fMRI NF training may have clinical implications with improved safety and minor side effects (31).

## Methods and analysis

### Aim

- To determine whether rt-fMRI neurofeedback of the goal-directed system could improve the depressive and/or anhedonic symptoms of depressed patients after training and at follow-ups;
- To examine the short-term and long-term effects of rt-rMRI neurofeedback on the goal-directed RL behavioral and/or connectivity imaging mediators.

### Participants

The study was approved by the ethical review committee at the Army Medical University (AMU, PRC) and conformed to the Declaration of Helsinki. The study has been submitted to the Chinese Clinical Trials Registry (ChiCTR, https://www.chictr.org.cn/). The trial identifier will be made available upon approval of the registration. After signing the informed consent form, the clinical data and general information of all participants will be collected, including age, gender, duration of illness, years of education, and medication history. The clinical diagnosis and symptom assessment will be completed by psychiatrists according to DSM-5 combined with a Mini-international Neuropsychiatric Interview (MINI). We will use the Hamilton Depression Scale-17 (HAMD-17) and the Montgomery–Åsberg Depression Rating Scale (MADRS) to assess anhedonia symptoms. Participant selection and recruitment processes were not conducted prior to the submission of the study.

(1) High anhedonic depression group. Inclusion criteria: ① diagnosed with major depression, with at least one core symptom (loss of pleasure or interest in all or nearly all activities) (MADRS item 8 ≥ 4) or non-reactive mood (items 1 or 2 ≥5, ② at least three following symptoms during the most severe phase of this depressive episode, including (a) significant psychomotor disturbance (HAMD items 8 or 9 ll1); (b) appetite/weight loss (HAMD items 12 or 16 = 2); (c) late insomnia (waking at least 2h earlier than usual) (HAMD item 6 ≥ 1); and (d) guilt (HAMD ≥ 1) (32); ③right-handedness. Exclusion criteria: ① comorbid with other mental disorders or drug or alcohol dependence; ② has received any previous antidepressant or antipsychotic medication (including benzodiazepines and other sedatives and hypnotics); ③ with severe physical diseases such as heart, liver, or kidney impairment, endocrine disorders, or infectious diseases; ④ substance dependence or abuse; ⑤ vision impairments; ⑥ pregnant or breastfeeding women; ⑦ MRI contraindications; and ⑧ currently receiving any emotional ability training.

(2) Healthy control group. Inclusion criteria: ① without a history of mental illness and family history of mental illness; ② age 18–55; ③ HAMD-17 < 7; and ④ right-handedness. Exclusion criteria: ① diagnosed with depression or other axis I diseases; ② has received any previous antidepressant or antipsychotic treatment (including benzodiazepines and other sedative and hypnotic drugs); ③ with severe physical diseases such as heart, liver, renal impairment, endocrine diseases, and infectious diseases; ④ substance dependence or abuse; ⑤ vision impairment; ⑥ women pregnant or breastfeeding; ⑦ MRI contraindications; and ⑧ currently receiving any emotional ability training.

### Procedure

**Figure 2:**
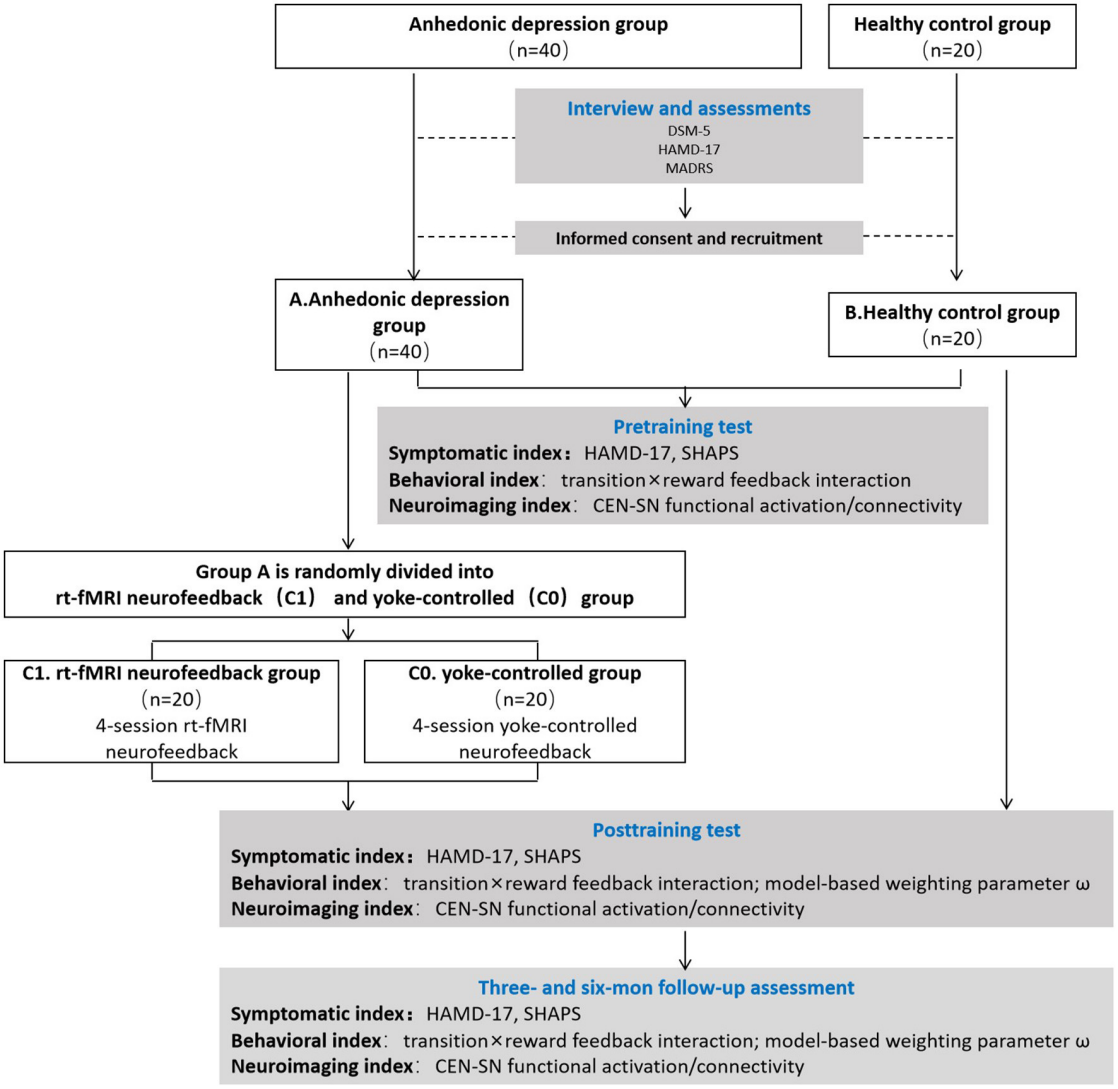
Consolidated Standards of Reporting Trials (CONSORT) flow diagram summarizing the study design.

This study is a double-blind randomized yoke-controlled rt-fMRI NF experiment, with follow-up assessments at 3 and 6 months after rt-fMRI NF (Figure 2). Participants will be randomly assigned to the experimental group (NF/YC) and given random numbers by a third party, who encodes the intervention with matching random numbers. The experimenter who will rate the outcome and/or analyze the data will be blind to group assignment. The CRED-NF checklist indicating which analyses will be performed and listed as supplementary material (available on the Open Science Framework preregistration, https://osf.io/ec396; CRED-NF checklist, https://osf.io/8wcqt).

#### Pre- and post-test

Before and after the neurofeedback intervention, the primary and secondary outcome measures of the depressive and anhedonic symptoms will be assessed in all subjects. The behavioral tasks will be repeated post-training to evaluate the NF training effect on goal-directed behavior. The follow-up assessments include the primary outcome of depression and secondary outcome of anhedonia, the goal-directed RL (model-based weighting parameter ω), and the CEN-SN functional connectivity (Pearson's correlation) based on prediction errors (PEs) (Figure 2).

##### Primary and secondary outcome measures

Primary outcome measures: HAMD-17 (33). The severity of depressive symptoms will be assessed using the total score of HAMD-17. The higher the score, the more severe the depressive symptoms.

Secondary outcome measures: Snaith–Hamilton Pleasure Scale (SHAPS) (34). The scale includes four aspects of interest/entertainment, social interaction, sensory experience, and food/drink, and has a total score of 14 to 56. It has good validity and reliability to evaluate the hedonic experience of adult outpatients with MDD (35).

The changes in depression and anhedonia symptoms will be assessed using HAMD-17 and SHAPS before and after the NF intervention, respectively. Follow-up assessments of depression (HAMD) and anhedonia (SHAPS) symptoms will be conducted at 3 and 6 months after the intervention.

##### Behavioral task

The reward learning task (two-stage Markov decision task) (30) will be adopted. All participants will practice an 8-min Markov decision task outside of the scanner room before formal NF training, for which instructions will be provided to the participants. After practice, the participants will rest for 5 min before completing the formal NF training. The practice and formal tasks will be presented using MATLAB 2019a (https://www.mathworks.com) and Psychtoolbox 3.0 (http://psychtoolbox.org).

**Figure 3:**
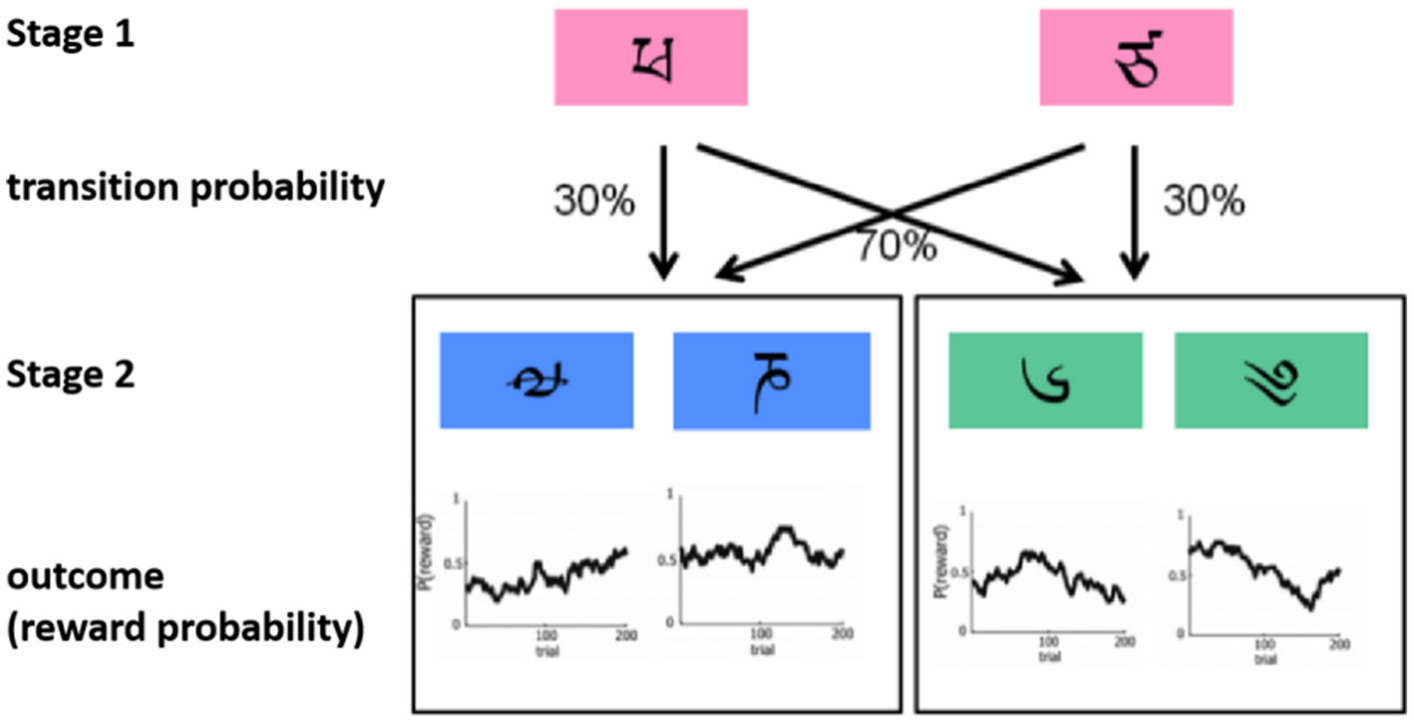
Schematic diagram of the Markov reward decision task adapted from Daw et al. (30).

The task will be divided into two stages. In stage 1, participants choose one of two stimuli (red) (stage 1, left panel), which leads to the second stimulus (green) in 70% of the cases and to the third stimulus (blue) (stage 2) in 30% of the cases. The other stimulus (stage 1, right panel) leads to the second stimulus (green) in 30% of the cases and to the third stimulus (blue) in 70% of the cases. The six stimuli at the two stages will be selected from the alphabets that are unfamiliar to the participants to encourage progressive learning. The possibility that the stimulus of stage 1 changes to the second stage through the key press of participants is called the transition probability (common transition 70% vs. rare transition 30%). The selection of experimental stimuli in stage 2 may result in either a reward or no reward. To motivate participants to continue learning, the reward will follow a slowly drifting Gaussian random walk with probability changes ranging from 0.25 to 0.75 (Figure 3).

**Figure 4:**
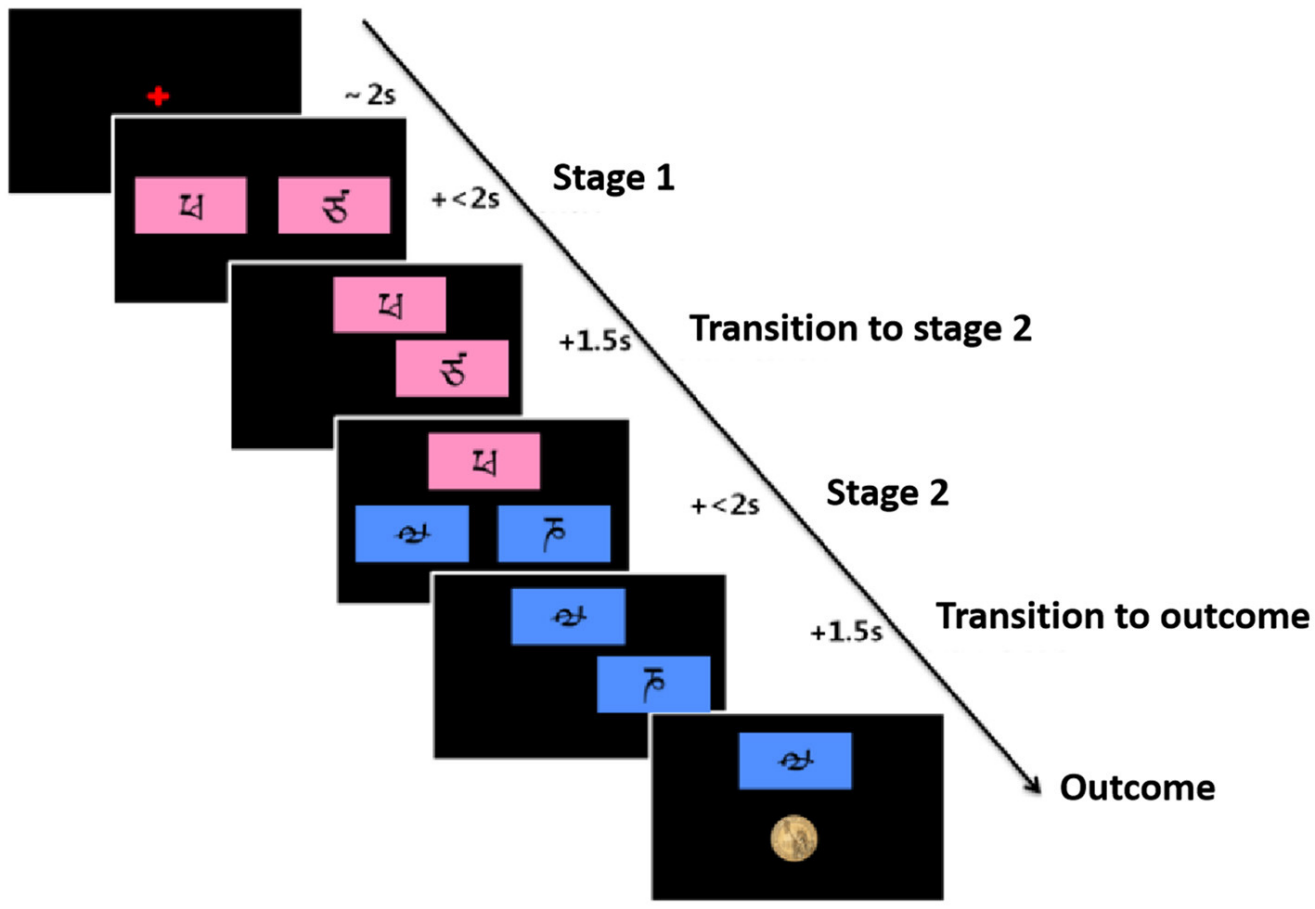
Flowchart of the Markov reward decision task, adapted from Daw et al. (30).

Participants will be encouraged to make options to receive as many rewards as possible and will be informed prior to practice and formal experiments that all rewards will be paid off at the end of the experiment. The task flow is as follows (Figure 4).

#### Real-time fMRI neurofeedback of individual goal-directed system in anhedonic depression

The anhedonic depressed participants (*n* = 40) will be randomized into the neurofeedback (NF) group (*n* = 20) and the yoke control (YC) group (*n* = 20). We used blocked randomization to form the allocation list (1:1 ratio) for the two comparison groups (NF and YC), by using a computer random number generator to select random permuted blocks (size = 10). The NF group will receive rt-fMRI neurofeedback intervention, with ~60 min per session, once per week for a total of four sessions. Participants in the YC group who are paired with one specific participant from the NF group will receive feedback signals from that participant during the same run. Thus, for the YC group, the sham rt-fMRI signals will not be corresponding to the participant's brain activity. The healthy control (HC) group (*n* = 20) will receive no intervention.

##### Sample size

The sample size is determined based on a heuristic justification (36) according to median recruits (*n* = 18) of fMRI neurofeedback (37). Moreover, given that this is a feasibility study with constraints on limited resources, a power calculation is not necessary according to the reporting and experimental design consensus in the NF field (38).

##### Real-time magnetic resonance imaging

MR images will be collected using 3.0T Siemens (Trio, Erlangen, Germany) with a 32-channel head coil. T1-weighted structural images will be collected from the whole brain with the magnetization-prepared rapid gradient echo (MPRAGE) sequence for anatomical localization of functional images. FOV = 256 × 256 mm, 176 sagittal slices, layer thickness = 1.0 mm, resolution = 1.0 mm^2^, TR/TE = 1900/2.52 ms, and flip angle = 9°. Echo-planar imaging (EPI) T2^*^-weighted functional images will be collected across the whole brain using a gradient echo planar imaging (EPI) sequence, FOV = 192 × 192 mm, matrix size = 64 × 64, and spatial resolution = 3 × 3 × 3 mm^3^, flip angle = 90°, and TR/TE = 2000/30 ms. To reduce the head motion, a strip of medical tape will be attached horizontally over the forehead of the participant and be spread to both sides of the head coil so that the participant could feel the head motion subjectively to prevent it from exceeding the range of head motion (39).

Real-time functional imaging will be achieved by using a customized function during MR image reconstruction, with TurboExport (Brain Innovation, Maastricht, The Netherlands) to convert input pixel data for each volume into images. Each image generated will be preprocessed in real time using the Turbo-BrainVoyager (Brain Innovation, Maastricht, The Netherlands). Preprocessing includes motion correction (by realigning each image to the first image of the session) and spatial smoothing (Gaussian kernel of half-height and 5 mm in full width). The computer presenting the task will communicate with Turbo-BrainVoyager via Transmission Control Protocol (TCP) to acquire preprocessed real-time data to be coregistered to the localizer anatomical image (first session) and generate feedback displays to the participants.

##### Network-based rt-fMRI NF intervention

The network-based rt-fMRI NF intervention of each session will be divided into 6 runs (5 NF runs and 1 transfer run), at 10 min/run. Subjects will be asked to raise or lower the height of a progress bar on the screen through cognitive effort. The height of the bar will depend on the signal intensity of regions of interest (ROIs) in the CEN and SN measured on each trial. Participants will be asked to regulate their brain signals through internal thought without specific instructions, with the feedback signaling the balance between the average signal intensities of the CEN and SN. They will know neither the source nor the calculation of the feedback signal. Before each session, participants will be explicitly instructed to try to avoid movement, including facial expressions, limb movements, and irregular breathing patterns.

During the scan, anatomical images will be recorded first (~5 min) and preprocessed immediately after image reconstruction based on a separate computer using BrainVoyager. After that, the results will be transmitted to Turbo-BrainVoyager to obtain real-time functional data aligned to the target ROIs of neurofeedback. Each run will start with a rest phase, during which the baseline and change in the feedback signal will be calculated. The feedback will be based on the differential signal between the mean values of all the voxels in the individualized ROIs in the CEN and SN. The baseline for this difference signal will be defined as the median difference between the initial resting signal of the SN and the CEN ROIs, and the lower and upper limits will be set to be two standard deviations from this baseline. The upper and lower limits will be updated to the average of the five lowest/highest median differences of this run before each block (29). The regulatory success targets the larger differential signal between CEN and SN. The transfer run will test the generalization of NF training without feedback signals during which the participants will be required to regulate their brain signals in the same way as the NF training run (40).

After each run, the participants will appraise their control over the progressive bar height (0–10 points) and their cognitive difficulties (0–10 points) with handles. Between runs, the participants will be allowed to rest for 5 min without significant head movement. After the NF intervention outside the scanner, the participants will write down the strategies they used to regulate their brain signals (e.g., through internal thought) and evaluate the effectiveness of strategies (0–10 points). To evaluate the demand characteristics, they will also indicate which group (NF or YC) they believe they belong to.

### Statistical analysis

#### Behavioral data

##### Statistical analysis

IBM SPSS Statistics 22 (https://www.ibm.com/spss) will be used. The 2 (groups: high anhedonia group and healthy control group) × 2 (transition conditions: common transition and rare transition) × 2 (reward feedback: reward and non-reward) factor mixed design will be conducted. The general linear mixed model (GLMM) will be used to investigate the main effects and interactions of the group, transition condition, and reward feedback on choice behavior. The interactions of transition conditions and reward feedback will represent the effect of the goal-directed system on the choice behavior (goal-directed RL), and the main effect of reward feedback will represent the effect of the habitual system on the choice behavior (habitual RL). The three-way interaction of the group, transition condition, and reward feedback will be examined to test for group differences in goal-directed RL. The two-way interaction of group and reward feedback will be examined to test for group differences in habitual RL.

##### Computational modeling

MATLAB 2019a (https://www.mathworks.com/) and the Reinforcement Learning algorithm will be used to model the behavioral data and estimate the learning rate α, inverse temperature parameter β, repetition parameter p, and model-based learning weight ω of individual subjects.

① value estimates. The state-action values for the first and second stages of each trial are determined by the weighted sum of the model-based action value Q_MB_ and the model-free action value Q_MF._ The weighted sum is expressed as follows.


(1) $$\begin{array}{l} Q_{net}\left(S_{A},a_{j}\right)=\omega Q_{MB}\left(S_{A},a_{j}\right)+{\left(1-\omega \right)Q}_{MF}\left(S_{A},a_{j}\right) \end{array}$$

It is assumed that individual behavioral choice in the first stage is the weighted sum of goal-directed and habitual learning, with the weight of ω (0~1). If ω is close to 0, the choice mainly depends on habitual learning; if ω is close to 1, the choice mainly depends on goal-directed learning.

(a) Model-based state-action value:


(2) $$\begin{array}{l} Q_{MB}(S_{A},a_{j})=P(S_{B}|S_{A},a_{j})\underset{a\in \{a_{A},a_{B}\}}{\max}Q_{MF}(S_{B},a)\\ \;+P(S_{C}|S_{A},a_{j})\underset{a\in \{a_{A},a_{B}\}}{\max}Q_{MF}(S_{C},a) \end{array}$$

(b) Model-free state-action value:


(3) $$\begin{array}{l} Q_{MF}\left(S_{i,t},a_{i,t}\right)=Q_{MF}\left(S_{i,t},a_{i,t}\right)+\alpha_{i}\delta_{i,t} \end{array}$$

For the first and second stages, learning rates α_1_ and α_2_, respectively, represent the extent to which the reward results are used for learning.

(c) Prediction error:


(4) $$\begin{array}{l} \delta_{i,t}=r_{i,t}+Q_{MF}\left(S_{i+1,t},a_{i+1,t}\right)-Q_{MF}\left(S_{i,t},a_{i,t}\right) \end{array}$$

The action probabilities of the first and second stages of each trial are regarded as a softmax function of the inverse temperature parameter β, the repetition parameter *rep*, and the indicator function *p* as follows.


(5) $$\begin{array}{l} P(a_{i,t}=a|s_{i,t})=\frac{exp(\beta [Q_{net}(s_{i,t},a)+p\cdot rep(a)])}{\sum_{a^{\prime }}exp(\beta_{i}[Q_{net}(s_{i,t},a^{\prime })+p\cdot rep(a^{\prime })])} \end{array}$$

The inverse temperature parameters β_1_ and β_2_ represent the randomness of the first- and second-stage choices. The repetition parameter p and the indicator function represent the reproducibility of the choice, that is, the tendency to choose the same choice as in the previous trial, regardless of the actual value of the choice. Additionally, *p* > 0 represents choices the same as the previous trial, and *p* < 0 represents choices different from the previous trial.

The parameters of each subject will be estimated using the R software and its Stan toolkit with the Markov Chain Monte Carlo (MCMC) estimation which has been previously described (4). The model evidence of the Akaike information criterion (AIC) and the Bayesian information criterion (BIC) will be calculated separately to estimate the model fit of reinforcement learning to the behavior.

#### Imaging data

##### Preprocessing analysis

DPABI V7.0 software (http://rfmri.org/DPABIV7) will be used for data pre-processing, in which the DICOM data will be converted into NIFTI format. In order to remove the effects of subject maladaptation during the scan as well as the effects of inhomogeneous magnetic fields on the results, data from the first 10 time points will be removed. Then, time correction, head motion correction, image standardization, image smoothing, detrending, and filtering (0.01~0.08 Hz) will be conducted, as well as the removal of head motion parameters.

##### Task-state PE signal analysis

BOLD signal analysis of ROIs during the task will be performed using SPM12 (https://www.fil.ion.ucl.ac.uk/spm/software/spm12/). The PE and relative partial differential calculations in the action selection phase and the reward outcome phase will be included as regressors in the general linear model, with ω (see Equation 1) as a covariate (30). The standard hemodynamic function will be constructed for ROIs using MarsBaR (https://sourceforge.net/projects/marsbar/) with FDR correction for control of false discovery rate in voxelwise tests (41). The ROIs will be made with WFU_PickAtlas software (https://www.nitrc.org/projects/wfu_pickatlas) according to the activation map during MDT for PEs.

The ROIs within CEN/SN will be mapped to CEN and SN which are functionally defined according to 14 intrinsic connectivity networks (ICNs) that are comprised of 90 distinct ROIs (42, 43). The DMN, SN, and CEN will be constructed from network templates with the Group ICA of fMRI Toolbox (GIFT, http://mialab.mrn.org/software/gift/) for use in the constrained ICA. The differential functional image (CEN - SN strength) during the first session will be used to create individualized network masks as neurofeedback target regions during the subsequent sessions. The nuisance regressors including six motion parameters and temporal derivatives as well as their quadratic terms and 25 physiological noise components will be taken into consideration. The first two runs of the first session will be discarded without additional analysis. The time courses (extracted using Turbo-BrainVoyager online) of ROIs will be high-pass filtered (0.01 Hz/100 s) and normalized (z-transformed).

##### Self-regulation performance

For each subject in the patient group in each session, self-regulation performance will be assessed with the CEN-SN activation difference (computed through online time courses of ROIs extracted using Turbo-BrainVoyager) during the training. Self-regulation performance will be defined as the mean % signal change of ROIs (CEN and SN) over all the NF runs. The self-regulation performance will then be submitted to an ANOVA with the factors group (NF vs. YC) and session (four sessions) for the group and/or time effects of rt-fMRI NF training.

Additionally, to investigate the pre- and post-training difference in CEN-SN functional connectivity for each patient group, we will apply the paired *t*-tests or Wilcoxon signed-rank tests (depending on parametric or non-parametric distributions) between the pre- and post-training tests, while undergoing MDT behavioral task during neuroimaging.

#### Intervention effect analysis

##### Primary and secondary outcomes

We will examine the difference in depressive symptoms between the NF and YC groups at the post-test after the training. Bayesian ANCOVA with the group as a fixed factor and the baseline HAMD scores as covariates will be used, using the default JASP (https://jasp-stats.org/) multivariate Cauchy priors (44), and the statistical approach has been described previously (25). The advantage of ANCOVA over repeated-measures (RM) models has been mentioned elsewhere (45). The additional covariates include age, gender, and duration of illness. The dependent variable will be post-test HAMD scores. Similar analyses will be performed on the anhedonic symptoms (SHAPS), with the same covariates and dependent variable. Additionally, similar analyses will also be performed on the primary and secondary outcomes at 3- and 6-month follow-ups.

##### GD-RL behavioral and connectivity imaging indicators

The behavioral (model-based weighting parameter ω) and connectivity imaging indicators (CEN-SN activation difference) will be used as potential mediating factors to determine whether behavioral and imaging indicators could explain the changes in depressive and/or anhedonic symptoms. Within-group explorative analysis will be conducted using a stepwise regression using a bootstrapping test when multiple mediators are concerned (46) with PROCESS macro (http://www.afhayes.com/) of SPSS 22.

## Discussion

The deficits in RL represent a sub-phenotype of anhedonia, with partially separable neural substrates (47). The decision-theoretic view of depression favors the compromise of the GD system, rather than the habitual system in depression (48), the former of which is present in both behavioral and neural manifestations during RL for clinical and subclinical depression (5, 7, 49). Acute stress affects the allocation of neural resources within large-scale brain networks, especially the balance between the CEN/SN in the GD system due to cognitive demands (50). Additionally, the acute stress (during the last month) is supposed to trigger shifts in goal-directed vs. habitual behavior pattern, which is concomitant with specific network configurations, especially frontostriatal circuits (CEN: LPFCal SN: VTA/putamen/caudate↓, lateral/medial OFC↓) (7). Stronger PEs may contribute to highly variable momentary mood fluctuations due to stronger reward feedback dynamics, whereas the influence of PEs on RL is compromised in depressed individuals (51). In summary, these studies highlight a promising target for RL-based NF interventions for anhedonic depression to produce flexible mood changes. Moreover, the focus on the GD-RL and imaging substrates in the current protocol may provide more insights with regard to the factors driving NF outcomes (38).

The novelty of the current study is that we focus on neither single ROI nor functional connectivity limited to a few ROIs, but rather the balance between two functionally intertwined brain networks (CEN/SN), and this approach has been utilized in self-regulation and sustained attention (27, 29). However, the impact of network-based NF on reward learning is still unknown. Thus, we propose to simultaneously decrease CEN and increase SN activity (CEN: LPFC↓; SN: VTA/putamen/caudate↑, lateral/medial OFC↑), which could potentially improve mood and its fluctuations. Individualized NF training based on a goal-directed subnetwork is potentially advantageous for improving treatment response to anhedonia symptoms for depressed patients.

Additionally, the current study takes into account the potential confounding factors of motivation and demand characteristics. The mechanism underlying NF may come from NF-specific or non-specific contextual factors as well as general non-specific effects such as practice, placebo, and natural change (cognitive development or decline)(38). Therefore, a double-blind yoke-controlled design is adopted in this study to control for individual differences in motivation and effort. As a limitation, insufficient power may result from the limited sample size. To control for inter-subject variance, yoke-controlled NF will be provided for the participants to modulate the fMRI signals, in which sham feedback has been shown to regulate neural activity in cognitive control compared to passively viewing conditions (52).

The caveat of this study is the use of CEN and SN based on intermittent, rather than continuous imaging feedback signals, due to the signal calculation speed of network parameters and the high cognitive load of momentary feedback (53). Accumulating evidence indicates that intermittent feedback is more efficient than continuous feedback to reinforce the participants' internal thoughts (54, 55). However, other studies showed that continuous feedback improved regulation ability (56). Additionally, monetary reward elicits a greater extent of self-regulation than non-reward feedback (57). Therefore, further study would be able to determine the influence of intermittent vs. continuous feedback and trial-by-trial virtual monetary reward on GD-RL, though the monetary reward in this study is not contingent on participants' decision as in the Markov decision (MD) task. Feedback design for NF training should be carefully considered in future research.

Finally, accumulating evidence converges on the feasibility and effectiveness of frontostriatal connectivity as NF targets (58). Within SN, the lateral and medial part of OFC as well as ACC densely project to the dorsal (e.g., caudate) and ventral striatum, which are involved in goal-directed control of instrumental actions (59). Moreover, the clinical efficacy of NF targeting both emotion areas and higher visual areas may indicate the non-specific training effects of NF, e.g., the brain control and reinforcement components (25). Therefore, confounds from the non-specific training effects should be taken into consideration during the explanation of the results. Additionally, further empirical studies and systematic reviews may shed light on the most efficient NF targets and provide more evidence on NF targeting specific ROIs of CEN/SN and connectivity-based and network-based NF within the GD-RL systems.

## Ethics statement

The study was reviewed and approved by the ethical review committee at the Army Medical University (AMU, PRC). Written informed consent to participate will be obtained from participants.

## Author contributions

XW: Conceptualization, Data curation, Funding acquisition, Methodology, Writing—original draft. XZ: Data curation, Methodology, Resources, Writing—review & editing. JL: Data curation, Methodology, Resources, Writing—review & editing. YG: Formal analysis, Software, Visualization, Writing—review & editing. ZF: Supervision, Writing—review & editing, Resources.
